# A Guide for Caring for Patients Amidst the Novel Coronavirus Pandemic

**DOI:** 10.1017/dmp.2020.381

**Published:** 2020-10-13

**Authors:** Graham Brant-Zawadzki, Jonathan Boltax, Steven Bott, Matthew Chapman, Megan Fix, Andrew Freeman, Matthew Fuller, Stephen Hartsell, Neil Krulewitz, Holly Ledyard, Michael Morgan, Robert Stephen, Lucy Unger, Wesley Williams, Matthew A. Roginski, Erin Lingenfelter, Cole Sloan, Anna Ciullo

**Affiliations:** University of Utah Hospitals, Division of Emergency Medicine, Salt Lake City, UT; Dartmouth-Hitchcock Medical Center, Lebanon, New Hampshire

**Keywords:** COVID-19, guide, SARS-CoV-2

The speed and manner in which health care systems respond to a crisis, such as the coronavirus disease (COVID-19) pandemic, are critical. An effective response requires policies based on prior evidence and available facts, but also easily amenable to rapid recalibrations as new data and knowledge emerge. In March 2020, the University of Utah Hospital and Clinics recognized the escalating scale and scope of COVID-19 and its potential impact on our ability to provide optimal patient care. In the hopes that our efforts may help guide other institutions in their own and future responses, we present our response to this pandemic.

As part of a larger institutional response, a multidisciplinary coalition, including emergency medicine physicians, intensivists, and anesthesiologists, developed a best-practice guide to the acute management of COVID-19, entitled: The University of Utah COVID-19 Respiratory Management Guide.^1^ Our group designed this resource as a living document to be continuously and immediately updated as new data, guidelines, and resource availability (or lack thereof) become known. This guide offers clinicians an adaptable compilation of current best practices for the clinical evaluation and treatment of COVID-19. It further serves as a reference to policies and logistics developed to best facilitate that care within and between departments. We supplemented this guide with open-access video tutorials and simulations published on our University Intranet as well as in-person High-Fidelity Simulation training sessions held jointly with residents, attending faculty, mid-level providers, nurses, and respiratory therapists. Over an initial 4-month period from March 14 to July 15, 2020, our department treated over 402 patients who tested positive for COVID-19. Our designated Emergency Department respiratory unit treated an average of 20–30 patients per day. Following implementation of our protocols, 57 patients were intubated according to a protocol of which 17 presented with primary respiratory failure with no reported decline of intubation success rate.

*The University of Utah COVID-19 Respiratory Management and Critical Care Reference Guide* is an evolving and easily accessible clinical resource amidst a deluge of daily information and policy updates.

## Disclaimer Statement

The recommendations in this document are based on a mixture of expert opinion and the best available evidence of which we were aware at the time of publication. The reader must understand that the protocols, algorithms, and practice recommendations were and are developed amidst a dynamic and ever evolving response to and understanding of severe acute respiratory syndrome coronavirus 2 (SARS-CoV-2) and COVID-19. While the contributors are representatives of the University of Utah Hospitals and Clinics, the presented protocols are not official Hospital doctrine but rather the recommendation of the authors and contributors themselves.
